# Supplementary material for: A high-resolution mRNA expression time course of embryonic development in zebrafish
Source: eLife. 2017 Nov 16;6:e30860. doi: 10.7554/eLife.30860 (PMC5690287; doi:10.7554/eLife.30860)
Supplement: Supplementary file 6. [file elife-30860-supp6.zip › biolayout-clusters-files/Cluster218-genes.html]

Cluster218


# Cluster218: Genes

| Ensembl ID | Gene Name | Chr | Start | End | Biotype |
| --- | --- | --- | --- | --- | --- |
| ENSDARG00000091271 | ENSDARG00000091271 | 21 | 6289421 | 6312591 | protein\_coding |
| ENSDARG00000060620 | lig4 | 1 | 28463910 | 28467542 | protein\_coding |
| ENSDARG00000005590 | mtf2 | 2 | 10820999 | 10848832 | protein\_coding |
| ENSDARG00000069681 | pcgf6 | 13 | 28552164 | 28559267 | protein\_coding |
| ENSDARG00000078576 | setd5 | 6 | 19735474 | 19794919 | protein\_coding |
| ENSDARG00000077987 | zfat | 16 | 25427120 | 25445933 | protein\_coding |
